# Mind–Body Exercise, Cognitive Reappraisal, Brooding, and Psychological Distress Among Middle-Aged Women

**DOI:** 10.3390/bs16091596

**Published:** 2026-09-08

**Authors:** Lin Yan, Rong Li, Guodong Zhang, Wenbin Liu

**Affiliations:** 1 Department of Sports, Shanxi Agricultural University, Jinzhong 030801, China; 2 School of Physical Education, Shanxi University, Taiyuan 030006, China

**Keywords:** mind–body exercise, cognitive reappraisal, brooding, psychological distress, middle-aged women

## Abstract

Mind–body exercise may support psychological well-being, but the cognitive–emotional processes associated with psychological distress among middle-aged women remain unclear. This cross-sectional study examined cognitive reappraisal and brooding as potential indirect pathways linking mind–body exercise participation with psychological distress. In May 2026, 423 women aged 40–55 years who had regularly participated in mind–body exercise at least once per week during the previous three months completed a paper-based questionnaire. Confirmatory factor analysis and structural equation modelling were conducted, and specific indirect effects were estimated using bootstrap procedures. Greater mind–body exercise participation was associated with more frequent cognitive reappraisal (β = 0.430, *p* < 0.001) and lower brooding (β = −0.274, *p* < 0.001). Cognitive reappraisal was negatively associated with brooding (β = −0.309, *p* < 0.001) and psychological distress (β = −0.322, *p* < 0.001), whereas brooding was positively associated with psychological distress (β = 0.353, *p* < 0.001). Significant negative indirect associations were observed through cognitive reappraisal (effect = −0.0032), through brooding (effect = −0.0024), and through the serial sequence of cognitive reappraisal and brooding (effect = −0.0010), with all bootstrap confidence intervals excluding zero. These findings highlight cognitive reappraisal and brooding as potentially important cognitive–emotional processes associated with the relationship between mind–body exercise participation and psychological distress among middle-aged women, providing a basis for future longitudinal and intervention research.

## 1. Introduction

Depression and anxiety are frequently observed expressions of psychological distress, which has emerged as a major challenge to global health. Its effects are not limited to those experiencing such symptoms but also create considerable pressure for their families and health service systems. Recent estimates released by the World Health Organization indicate that nearly 332 million individuals are affected by depression across the globe. The reported prevalence among women is approximately one and a half times that observed among men (WHO, 2025). Women in midlife, approximately 40–55 years of age, may experience substantial psychological and social demands associated with this stage of life (Infurna et al., 2020). For some women, this period may also overlap with the menopausal transition (Infurna et al., 2020; Kuck & Hogervorst, 2024; Shadhiya & Sasikala, 2025). The menopausal transition can be accompanied by sleep difficulties that may persist and adversely affect daytime functioning and well-being (Baker et al., 2018; Groeger & Hepsomali, 2026), as well as changes in mood, anxiety, and depressive symptoms, although these experiences vary considerably across women (Garg & Munshi, 2025). More broadly, midlife may involve changing family relationships, efforts to rebalance work and personal life, parenting and caregiving responsibilities, and multiple co-occurring stressors (Thomas et al., 2018). Among women, greater role overload has been associated with poorer mental health, while work stress and aspects of the working environment have been linked to less favorable occupational outcomes during midlife (Glynn et al., 2009; Hardy et al., 2018). Given the multiple psychosocial and health-related challenges that may accompany midlife, identifying effective and accessible strategies to support psychological well-being among middle-aged women has become an important public health priority.

Mind–body exercise is a broad term for practices that combine purposeful physical movement with controlled breathing and inwardly focused attention, including awareness of bodily sensations and meditative concentration (Blomstrand et al., 2023; X. Zhang et al., 2021). Yoga, Tai Chi, Qigong, and Baduanjin are widely included in this category. Pilates is also commonly regarded as a mind–body practice because its exercises require concentration, postural control, coordinated breathing, and precise movement (Y. T. Wang et al., 2017; Wells et al., 2012). In addition to physical movement, these practices involve attentional regulation and self-management, which may contribute to psychological health. Existing studies provide evidence of the potential psychological benefits of mind–body practices. One randomized study showed that participants receiving individualized yoga in addition to routine treatment experienced greater reductions in depressive symptoms and general psychological distress than those receiving routine treatment only (de Manincor et al., 2016). Another clinical trial found that yoga practice was followed by reduced insomnia and improved quality of life in postmenopausal women (Afonso et al., 2012). A later systematic review with meta-analytic evidence further indicated that yoga may improve sleep-related outcomes in women experiencing sleep difficulties (W. L. Wang et al., 2020). Despite these encouraging findings, most previous studies have emphasized whether these activities produce psychological benefits, while considerably less attention has been given to the psychological mechanisms that may explain the association between participation and reduced distress.

Mind–body exercise extends beyond physical movement because participants are required to maintain awareness of their breathing, bodily sensations, and inner experiences throughout the practice (Clark et al., 2015; Kouhbanani et al., 2023; Mehling et al., 2011). These attentional features may influence not only physical performance but also how people notice, understand, and manage stressful or emotionally meaningful situations (Lazzarelli et al., 2024). From the perspective of emotion regulation, cognitive reappraisal and brooding are two different psychological processes that may help explain this association. Cognitive reappraisal refers to reframing a situation before the emotional response is fully developed, thereby changing how strongly the event is experienced emotionally (Gross, 1998, 2002). It is often viewed as an adaptive approach because it enables individuals to consider stressful events from a different or more balanced perspective. However, its usefulness may vary depending on the nature of the situation (Gross & John, 2003; McRae & Gross, 2020). Brooding, by comparison, is an unhelpful form of rumination in which individuals repeatedly focus on their distress, perceived problems, and the gap between their present situation and personal expectations (Nolen-Hoeksema et al., 2008). Thus, reappraisal involves constructing a different understanding of an emotional experience, whereas brooding keeps attention fixed on negative thoughts without necessarily leading to effective solutions. Previous reviews and meta-analyses have accordingly described reappraisal and rumination as distinct emotion-regulation patterns related to emotional experience, psychological adjustment, and mental-health difficulties (Aldao et al., 2010; Watkins & Roberts, 2020).

Research into the psychological pathways of mind–body exercise remains fragmented, although cognitive reappraisal and brooding offer a useful framework for examining these processes. Existing studies can be broadly grouped into three related areas. The first area has emphasized general self-regulatory resources, such as attentional control, mindfulness, awareness of bodily and internal signals, positive affect, self-compassion, and self-control (Gard et al., 2014; Park et al., 2021; Riley & Park, 2015; Tihanyi et al., 2016). This literature has helped explain why mind–body practices may promote psychological health. However, these broad concepts provide limited insight into how people reinterpret stressful events or continue to dwell on negative thoughts. The second area has focused more directly on reappraisal and rumination. For example, women with greater yoga experience have reported using cognitive reappraisal more often than those with less experience (Kobylinska et al., 2018). Research involving mindfulness-based yoga and Bikram yoga among women with depression has also considered rumination as a psychological factor associated with, or potentially involved in, changes in depressive symptoms (La Rocque et al., 2021; Miao et al., 2023; Schuver & Lewis, 2016). Nevertheless, reappraisal and rumination have usually been examined in different studies. Much of the available evidence has also come from clinical populations or research limited to yoga. In addition, previous studies have commonly measured overall rumination rather than brooding, which represents its more harmful form.

A third area of research has begun to examine several psychological processes within the same indirect pathway. Among older adults, one study investigated whether social support and psychological resilience jointly explained the association between mind–body exercise and quality of life (Yang et al., 2024). Another study involving middle-aged and older participants examined emotion regulation and sleep quality as successive explanatory factors in the relationship between mind–body exercise and anxiety (Zheng et al., 2026). Related yoga research has also examined multiple psychological processes within the same explanatory framework. Tellhed et al. (2019) identified yogic breathing and mindfulness as relevant indirect processes associated with psychological health outcomes, while Parkinson and Smith (2022) examined several psychological factors, including mindfulness, self-compassion, interoceptive awareness, and emotion dysregulation, in relation to yoga experience and psychological well-being. These studies reflect increasing interest in connected psychological mechanisms, but they do not clarify whether specific adaptive and maladaptive cognitive and emotional processes are interconnected within the association between mind–body exercise and psychological distress. In particular, it remains unclear whether cognitive reappraisal and brooding function only as separate correlates of mind–body exercise or whether greater reappraisal is also associated with lower brooding within the same structural model. It therefore remains unclear whether greater participation in mind–body exercise is related to more frequent cognitive reappraisal, reduced brooding, and consequently lower psychological distress among women aged 40–55 years.

Accordingly, drawing on the theoretical and empirical literature reviewed above, the present study aimed to examine the associations among mind–body exercise participation, cognitive reappraisal, brooding, and psychological distress in women aged 40–55 years. Specifically, the proposed model examined whether greater participation in mind–body exercise was associated with more frequent cognitive reappraisal and lower brooding, whether cognitive reappraisal and brooding were associated with psychological distress, and whether cognitive reappraisal was related to brooding within the same structural model. Beyond these direct associations, the study further examined whether mind–body exercise participation was indirectly associated with psychological distress through cognitive reappraisal, through brooding, and through a serial pattern involving cognitive reappraisal and brooding.

This study contributes to the existing literature in two main ways. First, by examining cognitive reappraisal and brooding within the same structural model, it distinguishes between two conceptually different but potentially interconnected cognitive and emotional processes. Cognitive reappraisal represents an adaptive tendency to reinterpret emotionally meaningful situations, whereas brooding reflects a maladaptive tendency to remain focused on distress and unresolved negative thoughts. Considering these processes together makes it possible to examine not only their distinct associations with psychological distress but also their relationship with one another. In this respect, the study provides a more differentiated account of the cognitive and emotional processes associated with mind–body exercise than research that considers these processes separately or relies primarily on broader regulatory constructs. Second, the study extends previous research by focusing on women in midlife who participate in several forms of mind–body exercise and by examining general psychological distress rather than a single disorder or symptom. Together, these features broaden the understanding of the psychological correlates of mind–body exercise participation among women during midlife.

## 2. Literature Review and Hypothesis Development

### 2.1. Mind–Body Exercise, Cognitive Reappraisal and Brooding

Mind–body exercise integrates physical movement with breath regulation, sustained attention, and awareness of bodily and internal experiences. Empirical evidence directly linking mind–body exercise to habitual cognitive reappraisal remains limited, but several studies provide relevant evidence across different forms of mind–body practice. In a large cross-sectional study conducted during the COVID-19 lockdown, Sahni et al. (2021) found that yoga practitioners reported significantly more frequent use of cognitive reappraisal than both non-practitioners and individuals engaged in other spiritual practices. Long-term yoga practitioners also reported greater use of reappraisal than beginners. However, the study defined yoga practice broadly to include asana, breathing practices, meditation, and related yogic activities, and its cross-sectional design did not permit causal conclusions.

Related evidence has also emerged from studies examining broader emotion-regulation capacities rather than habitual cognitive reappraisal specifically (Gust & Bryan, 2024; Patel et al., 2018; Wadden et al., 2018). Daly et al. (2015) found in a randomized controlled trial that high-school students assigned to a yoga program showed greater improvement in overall emotion-regulation capacity than those participating in standard physical education. Research on Tai Chi has produced related findings that Tai Chi practitioners demonstrated stronger task-based emotion-regulation ability than demographically matched controls (Liu et al., 2018; S. Wang et al., 2025), while a 12-week Tai Chi intervention among college students was associated with improvements in working-memory capacity and emotion-regulation performance (Y. Wang et al., 2023). These latter studies assessed general or task-based emotion regulation rather than habitual cognitive reappraisal specifically. Although these studies do not directly assess habitual cognitive reappraisal, they provide complementary evidence that mind–body practices may be associated with regulatory capacities relevant to the reinterpretation and management of emotional experiences. Considered together with the more direct evidence concerning yoga and cognitive reappraisal, these findings provide a basis for examining whether greater participation in mind–body exercise is associated with more frequent use of cognitive reappraisal. Based on the literature, this study proposes the following hypotheses:

**Hypothesis** **1** **(H1).** *Mind–body exercise has a positive and significant association with cognitive reappraisal.*

Brooding represents a particularly maladaptive component of rumination and is characterized by passive and repetitive attention to distress, personal difficulties, and discrepancies between one’s current and desired circumstances (Nolen-Hoeksema et al., 2008). Research examining mind–body exercise and repetitive negative thinking has yielded more mixed findings (La Rocque et al., 2021). In a randomized controlled mixed-methods study involving women with major depression, Kinser et al. (2013) observed a distinctive trend toward reduced rumination following an eight-week gentle Hatha yoga intervention. A small one-year follow-up study subsequently suggested that exposure to the intervention might be associated with sustained improvements in rumination and other mental-health outcomes, although the very small follow-up sample substantially limited the generalizability of the findings (Kinser et al., 2014). Other controlled studies have produced less supportive results. In an investigation of 110 adults with persistent depression, West et al. (2021) found that yoga improved some facets of mindfulness relative to health education but did not produce a significantly different trajectory of rumination. Similarly, Vollbehr et al. (2023) reported that a single 20 min mindful-yoga session did not reduce rumination following a dysphoric mood induction relative to a relaxation condition; in that study, the stretching condition showed lower rumination instead. These findings indicate that the relationship may depend on factors such as intervention duration, repeated practice, comparison condition, and participant characteristics. Importantly, these studies generally assessed rumination as a broad construct rather than brooding as its more maladaptive component. A theoretical basis for expecting a negative association can also be derived from the attentional characteristics of mind–body exercise. These practices typically require sustained awareness of breathing, bodily sensations, movement, and present experience (Clark et al., 2015; Kouhbanani et al., 2023). Brooding, in contrast, involves persistent attention to distress, perceived difficulties, and unresolved negative thoughts. The different attentional orientations represented by these processes therefore provide a theoretical basis for expecting greater mind–body exercise participation to be associated with a lower tendency toward brooding. Based on the literature, this study proposes the following hypotheses:

**Hypothesis** **2** **(H2).** *Mind–body exercise has a negative and significant association with brooding.*

Cognitive reappraisal and brooding should not be understood simply as opposite ends of a single continuum. Cognitive reappraisal involves changing the meaning assigned to an emotional situation, whereas brooding involves repeatedly maintaining attention on negative feelings and self-relevant difficulties. Nevertheless, the two processes may be related through their shared dependence on cognitive control over negative information (Barkus, 2020). Successful reappraisal requires individuals to disengage from an initial interpretation, inhibit or update emotionally negative representations, and construct an alternative meaning (Ford et al., 2017; Kobayashi et al., 2020). By contrast, difficulties inhibiting negative information may make such reinterpretation more difficult while allowing repetitive negative thought to persist. Cohen et al. (2014) reviewed correlational and experimental evidence linking impaired inhibition of negative information with both reduced reappraisal and greater rumination, proposing inhibition as a cognitive process shared by the two strategies. Daches and Mor (2015) further found that brooding moderated the relationship between reappraisal and the inhibition of negative information, indicating that the effectiveness of reappraisal-related cognitive control may depend on an individual’s tendency to brood. In addition, Pe et al. (2013) demonstrated across trait and daily-life analyses that the emotional consequences of both reappraisal and rumination varied according to the ability to update emotional information in working memory. Although direct research examining the relationship between habitual cognitive reappraisal and brooding remains limited, the available evidence suggests that the two processes may be linked through cognitive control over negative emotional information. Greater use of cognitive reappraisal may therefore be associated with a lower tendency to remain engaged in brooding. However, the existing evidence does not establish the temporal direction of this relationship, and reverse or reciprocal associations remain possible. Based on the literature, this study proposes the following hypothesis:

**Hypothesis** **3** **(H3).** *Cognitive reappraisal has a negative and significant association with brooding.*

### 2.2. Cognitive Reappraisal, Brooding and Psychological Distress

Cognitive reappraisal may help reduce psychological distress by changing how an emotionally important event is understood before the emotional reaction is fully formed. Experimental evidence indicates that reframing unpleasant stimuli can weaken the intensity of negative feelings (Cui et al., 2024; Dryman & Heimberg, 2018). For instance, Ochsner et al. (2002) reported that participants experienced less negative emotion when they interpreted aversive images from a different perspective. This process was accompanied by greater activity in prefrontal areas responsible for cognitive control and changes in brain regions involved in emotional processing.

Findings from everyday settings point in a similar direction. In a three-week diary investigation, Nezlek and Kuppens (2008) observed that greater daily use of reappraisal tended to coincide with more favorable emotional experiences and better psychological adjustment. However, the pattern differed according to whether participants were regulating positive or negative emotions. Evidence obtained under stressful conditions also supports the importance of reappraisal. Troy et al. (2010) showed that reappraisal ability changed the relationship between life stress and depressive symptoms in women. When stress levels were high, women with stronger reappraisal skills reported fewer depressive symptoms than those with weaker skills.

Consistent with these findings, a meta-analysis covering 48 studies and 21,150 participants indicated that reappraisal was related to better mental-health outcomes and to lower levels of depression, anxiety, and negative affect (Hu et al., 2014). Nevertheless, its benefits are not equally strong in every situation. Troy et al. (2013) reported that reappraisal was more beneficial when stressful circumstances were relatively difficult to control. Overall, frequent or effective use of reappraisal tends to correspond with fewer negative emotional symptoms and more favorable psychological functioning, although its value partly depends on the context in which it is used. Based on the literature, this study proposes the following hypotheses:

**Hypothesis** **4** **(H4).** *Cognitive reappraisal has a negative and significant association with psychological distress.*

Brooding may contribute to higher psychological distress because it keeps individuals mentally focused on unpleasant feelings, unresolved difficulties, and perceived failures without necessarily helping them find workable solutions. Research conducted in everyday settings suggests that persistent self-focused thought can strengthen negative emotions and make them last longer. Using experience-sampling data, Moberly and Watkins (2008) observed that periods of stronger ruminative attention tended to coincide with more intense negative affect. Their findings also suggested that rumination may extend emotional reactions across daily experiences.

Evidence separating brooding from reflective pondering further indicates that brooding is the more harmful form of rumination. Burwell and Shirk (2007) reported that brooding predicted later growth in depressive symptoms among adolescents, particularly girls, whereas reflective pondering did not show the same pattern. The psychological relevance of repetitive negative thought also extends beyond depression (Moulds & McEvoy, 2025; Spinhoven et al., 2018). McLaughlin and Nolen-Hoeksema (2011) showed that rumination partly explained the shared occurrence of depressive and anxiety symptoms, indicating that it may operate across different forms of emotional difficulty. Longitudinal research by Michl et al. (2013) similarly found that rumination helped account for the link between stressful experiences and later symptoms of anxiety and depression in both adolescent and adult samples.

Studies focused specifically on women provide additional support. Lucena-Santos et al. (2018) reported that women with stronger brooding tendencies also tended to experience more symptoms of depression, anxiety, and stress. Miranda and Nolen-Hoeksema (2007) further found that brooding was related to suicidal thoughts one year later in a community sample, although this relationship differed according to participants’ initial psychological conditions. Overall, the available evidence suggests that brooding is connected with both the continuation and later development of several forms of psychological difficulty rather than only one type of symptom. Based on the literature, this study proposes the following hypotheses:

**Hypothesis** **5** **(H5).** *Brooding has a positive and significant association with psychological distress.*

### 2.3. Hypothesized Indirect Associations

The preceding literature suggests that mind–body exercise is associated with cognitive reappraisal and brooding, which are in turn associated with psychological distress. These relationships provide the basis for considering cognitive reappraisal and brooding as potential explanatory processes in the statistical association between mind–body exercise participation and psychological distress. Building on the direct associations proposed above, the present study further considers whether these processes are involved in distinct and serial indirect associations.

Although mind–body exercise is not equivalent to mindfulness training, many forms of mind–body exercise involve sustained attention to movement and breathing, awareness of bodily experience, and repeated disengagement from distracting thoughts (Larkey et al., 2009). The Mindfulness-to-Meaning Theory proposes that present focused attention facilitates decentering from habitual stress appraisals, broadens awareness of contextual information, and creates opportunities for more flexible reappraisal of emotional experiences (Garland et al., 2015). Longitudinal research has provided support for the proposed relationships among attentional control, decentering, broadened awareness, and reappraisal (Garland et al., 2017; Hanley et al., 2021). Related evidence from mind–body practices also points to improvements in emotion regulation. Patel et al. (2018) found that participation in a yoga-based meditation program was accompanied by increased cognitive reappraisal, while Y. Zhang et al. (2019) reported improvements in implicit emotion-regulation ability following a mind–body exercise intervention incorporating mindfulness-based yoga. Evidence from longitudinal research further indicates that cognitive reappraisal is negatively associated with psychological distress, although such findings have been obtained in populations different from the present sample (Boyes et al., 2016). Therefore, these findings suggest that, to the extent that mind–body exercise engages attention and awareness processes relevant to emotion regulation, greater participation may be associated with more frequent cognitive reappraisal, which in turn may be associated with lower psychological distress. Accordingly, cognitive reappraisal may constitute one indirect pathway linking mind–body exercise participation with psychological distress. Based on this reasoning, the following hypothesis is proposed:

**Hypothesis** **6a** **(H6a).** *Mind–body exercise has a significant negative indirect association with psychological distress through cognitive reappraisal.*

The attentional characteristics of mind–body exercise may also be relevant to brooding. Mind–body exercise directs attention toward present bodily sensations, breathing, and movement and requires repeated disengagement from distracting thoughts, whereas brooding involves persistent attention to distress, personal shortcomings, and unresolved problems (Larkey et al., 2009; Treynor et al., 2003). Research on yoga provides some empirical support for the relevance of repetitive negative thinking in this context. A systematic review of mechanisms underlying body-oriented yoga for major depressive disorder identified rumination as one of the psychological processes examined in previous studies and reported evidence linking reductions in rumination with treatment outcomes, although the available evidence was limited and did not focus specifically on brooding (Meister & Juckel, 2018). More broadly, a systematic review examining rumination and psychological distress found that maladaptive forms of repetitive thinking, particularly brooding and intrusive rumination, were associated with greater distress (Beck et al., 2023). These contrasting attentional orientations provide a basis for expecting greater mind–body exercise participation to be associated with lower brooding. Because stronger brooding tendencies are also associated with greater psychological distress, lower brooding may represent a distinct indirect pathway through which mind–body exercise participation is associated with lower psychological distress. Accordingly, the following hypothesis is proposed:

**Hypothesis** **6b** **(H6b).** *Mind–body exercise has a significant negative indirect association with psychological distress through brooding.*

Cognitive reappraisal and brooding may also operate as connected rather than entirely independent processes. Cognitive reappraisal involves reconsidering the meaning assigned to an emotional experience, whereas brooding involves maintaining attention on distress and unresolved negative thoughts. Research on repetitive cognition suggests that persistent negative thinking can maintain the cognitive representation of stressful experiences and prolong their psychological consequences (Brosschot et al., 2006; Watkins, 2008). Evidence from research on mindfulness further suggests that adaptive and maladaptive emotion-regulation processes may jointly characterize associations with psychological outcomes. Mamede et al. (2022), for example, examined cognitive reappraisal and rumination as potential explanatory processes in the association between mindfulness and depression, anxiety, and psychological distress. More directly relevant to the proposed sequence, recent research using structural equation modelling found that facets of trait mindfulness were associated with depressive symptoms through sequential relationships involving greater cognitive reappraisal and lower brooding rumination (Maruthy et al., 2026). Although mindfulness and mind–body exercise are not equivalent constructs, these findings provide relevant evidence that attention and awareness processes, adaptive reinterpretation, and maladaptive repetitive thinking may be interconnected within the same statistical pathway. Accordingly, the following hypothesis is proposed:

**Hypothesis** **6c** **(H6c).** *Mind–body exercise has a significant negative serial indirect association with psychological distress through cognitive reappraisal and brooding.*

**Figure 1:**
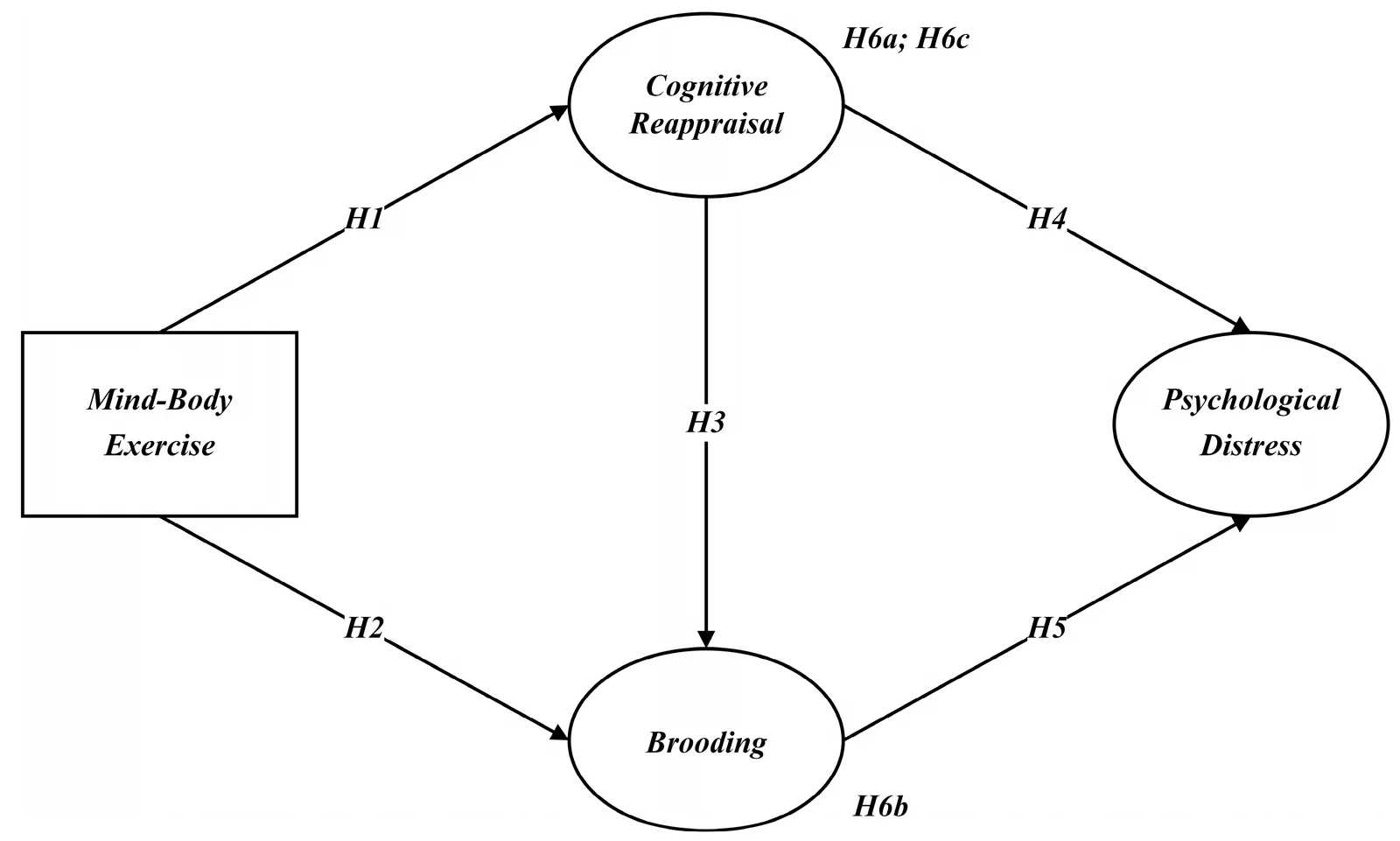
Hypothesis model.

All hypotheses are summarized in Figure 1.

## 3. Methodology

### 3.1. Participants and Procedures

This study adopted a cross-sectional survey design. Data were collected in May 2026 using a paper-and-pencil questionnaire. Participants were recruited from 18 community-based exercise sites in Shaanxi and Shanxi provinces, including community fitness centers, yoga and Pilates studios, and Tai Chi, Baduanjin, and Qigong activity groups. Potential participants who were present at these sites during the recruitment period were approached in person and invited to participate. Because convenience sampling was used, recruitment was not consecutive but depended on participant accessibility and eligibility.

The inclusion criteria were as follows: participants had to be aged between 40 and 55 years, have regularly participated in mind–body exercise (at least once per week) during the previous three months, such as yoga, Pilates, Tai Chi, Baduanjin, Qigong, or related activities, and be able to understand and complete the questionnaire independently. Participants who had previously practiced mind–body exercise but had not participated during the preceding three months were considered former participants and were not eligible. Participation in multiple modalities was not restricted; however, participants were asked to report only their primary mind–body exercise modality in the questionnaire. Before completing the questionnaire, participants were informed of the purpose of the study, the voluntary nature of participation, the anonymity of their responses, and their right to withdraw at any time. Informed consent was obtained from all participants. No personally identifiable information was collected, and all data were used only for academic research purposes.

A total of 500 paper questionnaires were distributed and returned. Following data screening, 77 questionnaires were excluded because of substantial missing responses, incomplete responses to the main study measures, or response patterns indicating insufficient data quality, such as highly repetitive or invariant responses. Because category-specific exclusion counts were not retained during the original screening process, a reliable retrospective breakdown of the 77 exclusions could not be provided. The final analytic sample therefore consisted of 423 participants, yielding a valid response rate of 84.6%. The questionnaire included demographic information, mind–body exercise-related items, and the main study measures.

Sample-size adequacy was considered in relation to the complexity of the proposed SEM. The final model included three reflective latent constructs—cognitive reappraisal, brooding, and psychological distress—measured by 17 observed indicators, while mind–body exercise participation was entered as an observed composite predictor. Five hypothesized structural paths were estimated. No formal a priori power analysis was conducted before data collection. The final analytic sample consisted of 423 participants and was considered sufficient for estimation of the proposed model.

As shown in Table 1, most participants were aged 45–49 years (58.4%). In terms of education, the largest proportion had a college or bachelor’s degree (48.2%), followed by high school or technical secondary school education (44.0%). Most participants were married or cohabiting (87.2%) and employed full-time (55.1%). Regarding the type of mind–body exercise, Pilates was the most commonly reported activity (37.4%), followed by yoga (32.9%).

### 3.2. Instruments

The questionnaire included demographic information and four main instruments. Demographic variables included age, education level, marital status, employment status, and type of mind–body exercise. The questionnaire was administered in Chinese. For measures originally developed in English, a translation and back-translation procedure was used. The items were first translated into Chinese and then independently back-translated into English. The back-translated versions were compared with the original items, and discrepancies were reviewed and resolved to ensure semantic consistency and cultural appropriateness.

Mind–body exercise participation was measured using three self-reported items adapted from the scoring logic of the Physical Activity Rating Scale-3 revised by Liang (1994). Participants reported their exercise intensity, average duration per session, and participation frequency during the past three months. A sample item was: “How often do you usually participate in mind–body exercise?” Each component was scored on five levels (1–5). Intensity ranged from very light to relatively high; duration was categorized as <10, 10–20, 21–30, 31–60, and >60 min per session; and frequency as <1/month, 1–3/month, 1/week, 2–3/week, and ≥4/week. Because intensity, duration, and frequency represent distinct components that jointly define exercise exposure rather than interchangeable reflective indicators of a single latent construct, mind–body exercise participation was treated as an observed composite exposure index rather than a reflective latent variable. Following the PARS-3 scoring approach, the overall score was calculated as intensity × (duration − 1) × frequency, yielding a possible range of 0–100 and retaining the original weighting of intensity, duration, and frequency. Higher scores indicated greater participation in mind–body exercise.

Cognitive reappraisal was assessed using the six-item cognitive reappraisal subscale of the original Emotion Regulation Questionnaire (ERQ) developed by Gross and John (2003). The original ERQ comprises 10 items assessing two distinct dimensions: cognitive reappraisal (six items) and expressive suppression (four items). Only the six cognitive reappraisal items were administered in the present study. The response format was adapted to a five-point Likert scale ranging from 1 = strongly disagree to 5 = strongly agree. A sample item was: “When I want to feel less negative emotion, I change the way I’m thinking about the situation.” Higher scores indicated greater habitual use of cognitive reappraisal.

Brooding was measured using the five-item brooding subscale of the Ruminative Responses Scale (Nolen-Hoeksema, 1991). The Chinese version retained the five-item structure and original response format. Participants rated how often they engaged in brooding thoughts when feeling down, sad, or depressed, from 1 = never to 5 = always. A sample item was: “Why can’t I handle things better?” Higher scores indicated higher levels of brooding.

Psychological distress was assessed using the six-item K6 scale developed by Kessler et al. (2002). All six items and the original 30-day reference period were retained. The response format was adapted for the present questionnaire to a five-point frequency scale ranging from 1 = never to 5 = always (rarely, sometimes, and often as the intermediate categories). Participants reported how often they had experienced each symptom during the past 30 days. A sample item was: “During the last 30 days, about how often did you feel hopeless?” Higher scores indicated greater psychological distress. In the present study, the K6 items were used to represent psychological distress as a continuous latent construct in the SEM; no diagnostic classification or established K6 cut-off scores were applied. Accordingly, the scores were not interpreted using conventional K6 clinical thresholds.

### 3.3. Data Analysis

Data analyses were conducted using SPSS 26.0 and AMOS 23.0. First, descriptive statistics and correlation analyses were performed using SPSS 26.0 to examine the basic characteristics and relationships among variables. Data were screened for missing values, outliers, and distributional assumptions before model estimation. Questionnaires with substantial missing data or incomplete responses to the main study measures were excluded during data screening, and no missing values remained in the final analytic sample. Univariate normality was assessed using skewness and kurtosis. The multivariate kurtosis value was 9.437 (critical ratio = 3.617), indicating a moderate departure from multivariate normality. Multivariate outliers were examined using Mahalanobis distance, and maximum likelihood estimation was retained.

Second, confirmatory factor analysis (CFA) was conducted in AMOS 23.0 to assess the measurement properties of the three reflective latent constructs—cognitive reappraisal, brooding, and psychological distress—while mind–body exercise participation was treated as an observed composite index. Models were estimated using maximum likelihood estimation. For model identification, one factor loading for each latent construct was fixed to 1.0, and the latent constructs were allowed to covary in the CFA. Third, structural equation modeling (SEM) was used to test the five hypothesized structural paths among the study variables.

**Figure 2:**
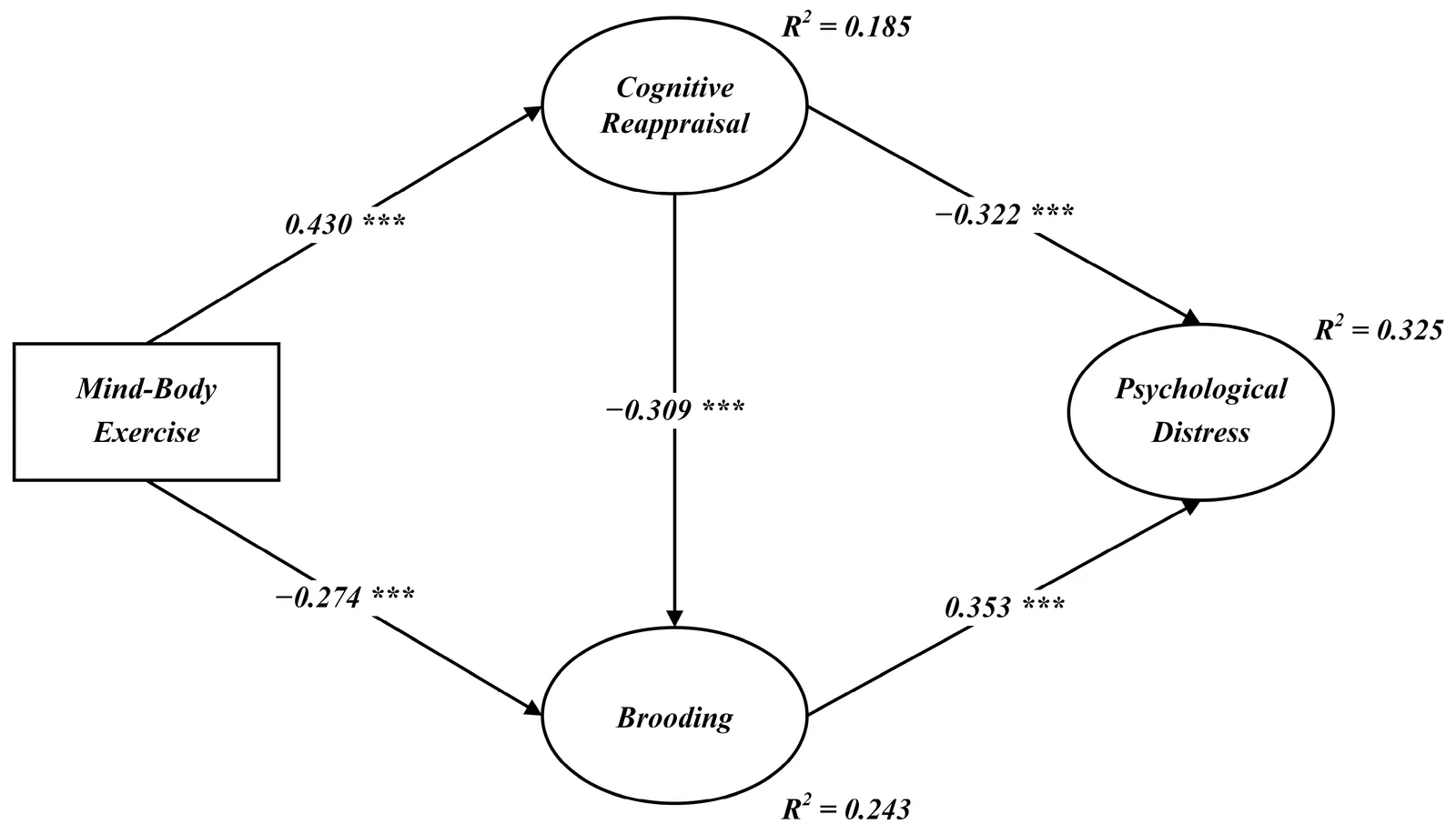
Structural model. Note: *** *p* < 0.001.

In addition to the SEM, a separate indirect-effects decomposition analysis was conducted using PROCESS Model 6 (Version 4.2) in SPSS 26.0 with 5000 bootstrap samples. In this supplementary analysis, the MBE composite score was entered as the predictor, cognitive reappraisal and brooding as sequential mediators, and psychological distress as the outcome. Composite scores for the psychological variables were used in the PROCESS analysis. Unlike the SEM presented in Figure 2, PROCESS Model 6 additionally estimated the direct mind–body exercise → psychological distress path to decompose the total association into direct and specific indirect effects.

Model fit was evaluated using χ^2^/df, GFI, AGFI, IFI, CFI, TLI, and RMSEA. Values of χ^2^/df < 3, GFI, AGFI, IFI, CFI, and TLI ≥ 0.90, and RMSEA ≤ 0.08 were considered indicative of acceptable model fit.

Common method variance (CMV) was assessed using Harman’s single-factor test. The results showed that the first unrotated factor explained 37.368% of the total variance, which was below the commonly recommended threshold of 50%. However, given the limitations of Harman’s single-factor test, this result was treated only as a preliminary diagnostic and does not rule out the possibility of CMV.

## 4. Results

### 4.1. Measurement Model

The CFA measurement model was evaluated independently before the structural model was estimated. Because mind–body exercise participation was treated as an observed composite exposure index, the measurement-model assessment was conducted only for the three reflective latent constructs: cognitive reappraisal, brooding, and psychological distress. The three-factor measurement model showed a good fit to the data: χ^2^ = 109.134, df = 116, *p* = 0.661, χ^2^/df = 0.941, GFI = 0.971, AGFI = 0.961, IFI = 1.002, CFI = 1.000, TLI = 1.002, and RMSEA = 0.000 (90% CI [0.000, 0.021]). The slightly above-unity IFI and TLI values can occur when χ^2^ is smaller than the model degrees of freedom and are consistent with the overall excellent model fit. These results supported the adequacy of the measurement model before evaluation of the structural relationships. As shown in Table 2, all standardized factor loadings ranged from 0.743 to 0.804. Cronbach’s alpha and composite reliability values ranged from 0.880 to 0.899, indicating good internal consistency. The average variance extracted (AVE) values ranged from 0.590 to 0.597, supporting convergent validity.

Discriminant validity was examined using the Fornell–Larcker criterion. As shown in Table 3, the square roots of AVE ranged from 0.768 to 0.773 and exceeded the corresponding inter-construct correlations. Discriminant validity was further assessed using the heterotrait–monotrait ratio (HTMT). The HTMT values were 0.429 for CR–BR, 0.468 for CR–PD, and 0.487 for BR–PD, all below the recommended threshold of 0.85, providing additional evidence of discriminant validity. Descriptive statistics and bivariate correlations for the MBE composite score and the three psychological constructs are also reported in Table 3. The MBE composite score had a mean of 31.31 (SD = 28.30), with an observed range of 0–100. Overall, the measurement model demonstrated adequate reliability, convergent validity, and discriminant validity.

### 4.2. Structural Model

The structural model showed a good fit to the data: χ^2^/df = 1.049, GFI = 0.965, AGFI = 0.955, IFI = 0.998, TLI = 0.998, CFI = 0.998, and RMSEA = 0.011. These results indicated that the proposed model adequately represented the observed data. As shown in Figure 2, the path analysis results were consistent with Hypotheses 1–5. Mind–body exercise was positively associated with cognitive reappraisal (β = 0.430, *p* < 0.001), consistent with H1. Mind–body exercise was negatively associated with brooding (β = −0.274, *p* < 0.001), consistent with H2. Cognitive reappraisal was negatively associated with brooding (β = −0.309, *p* < 0.001), consistent with H3, and negatively associated with psychological distress (β = −0.322, *p* < 0.001), consistent with H4. In addition, brooding was positively associated with psychological distress (β = 0.353, *p* < 0.001), consistent with H5. The model accounted for 18.5% of the variance in cognitive reappraisal, 24.3% of the variance in brooding, and 32.5% of the variance in psychological distress. Overall, these findings were consistent with the hypothesized structural associations among mind–body exercise, cognitive reappraisal, brooding, and psychological distress.

### 4.3. Indirect Effects Analysis

To supplement the latent-variable SEM, a separate indirect-effects decomposition analysis was conducted using PROCESS Model 6 with 5000 bootstrap samples. This supplementary analysis included a direct path from mind–body exercise to psychological distress, allowing the total association to be decomposed into the direct effect, total indirect effect, and three specific indirect effects. Accordingly, the direct mind–body exercise → psychological distress effect reported in Table 4 was estimated in the PROCESS analysis and was not included in the SEM presented in Figure 2. As shown in Table 4, in this supplementary analysis, the direct association between mind–body exercise and psychological distress was significant (effect = −0.0057, SE = 0.0015, 95% CI [−0.0087, −0.0027]). The total indirect effect was also significant (effect = −0.0066, BootSE = 0.0009, 95% CI [−0.0084, −0.0048]).

The three hypothesized specific indirect associations were all statistically significant. Consistent with H6a, mind–body exercise was negatively indirectly associated with psychological distress through cognitive reappraisal (effect = −0.0032, BootSE = 0.0007, 95% CI [−0.0046, −0.0019]). Consistent with H6b, a significant negative indirect association was also observed through brooding (effect = −0.0024, BootSE = 0.0006, 95% CI [−0.0035, −0.0014]). Finally, consistent with H6c, the serial indirect association through cognitive reappraisal and brooding was significant (effect = −0.0010, BootSE = 0.0003, 95% CI [−0.0015, −0.0005]). All three bootstrap confidence intervals excluded zero, providing support for H6a, H6b, and H6c.

The relatively small numerical values of these effects reflect their unstandardized scale. The magnitudes of the unstandardized indirect effects depend on both the measurement scale of the observed MBE composite score and the scaling of the latent psychological constructs. Therefore, the magnitude of these coefficients should be interpreted in relation to the metrics and scaling of the variables involved rather than compared directly with the standardized coefficients reported in the SEM. Given the cross-sectional design, these indirect effects are interpreted as statistical associations within the hypothesized model rather than evidence of temporal mediation.

### 4.4. Sensitivity Analysis

As shown in Table 5, adjustment for age, education level, and marital status did not materially alter the principal structural associations. All five hypothesized paths remained statistically significant (all *p* < 0.001), whereas none of the covariate paths was statistically significant. The adjusted standardized coefficients for both the hypothesized and covariate paths are reported in Table 5. These findings indicate that the principal structural results were robust to adjustment for the available sociodemographic characteristics.

## 5. Discussion

### 5.1. Theoretical Contributions

The present study makes several contributions to research on mind–body exercise and psychological well-being.

First, it extends research on psychological processes associated with mind–body exercise by identifying cognitive reappraisal and brooding as two specific cognitive–emotional processes associated with the relationship between mind–body exercise and psychological distress. Previous research has proposed that mind–body practices may influence psychological functioning through broad self-regulatory mechanisms involving attention, bodily awareness, interoception, and emotion regulation (Gard et al., 2014). Empirical studies have also examined mechanisms such as mindfulness, self-compassion, self-control, perceived social support, psychological resilience, and sleep quality (Park et al., 2021; Yang et al., 2024; Zheng et al., 2026). By focusing on cognitive reappraisal and brooding, the present study provides a more differentiated account of the cognitive processing of emotional experiences. The findings further showed significant indirect associations between mind–body exercise and psychological distress through cognitive reappraisal, through brooding, and through the serial sequence of cognitive reappraisal and brooding. These results therefore complement existing models based on broad regulatory resources by highlighting the potential relevance of both adaptive reinterpretation and maladaptive repetitive thinking.

Second, this study contributes to the emotion-regulation and rumination literature by examining cognitive reappraisal and brooding within the same structural model. Cognitive reappraisal and brooding are sometimes treated as broadly contrasting responses to negative emotional experiences, but they are conceptually distinct rather than opposite ends of a single continuum. Reappraisal involves altering the meaning assigned to an emotional situation, whereas brooding involves persistently focusing on distress, perceived shortcomings, and unresolved discrepancies. The present findings showed that cognitive reappraisal was negatively associated with brooding and that the two processes demonstrated different associations with psychological distress: reappraisal was negatively associated with distress, while brooding was positively associated with it. By incorporating the path from cognitive reappraisal to brooding, the study suggests that adaptive and maladaptive cognitive–emotional processes may be interconnected. More flexible reinterpretation of stressful experiences may coexist with a lower tendency to remain engaged in repetitive negative thinking, although the cross-sectional design does not establish the temporal or causal direction of this relationship.

Third, the study extends the population and outcome contexts in which psychological processes associated with mind–body exercise have been examined. Previous studies of psychological processes related to mind–body exercise have frequently focused on yoga-specific interventions, clinical populations, younger adults, older adults, or mixed samples of middle-aged and older participants. Recent research, for example, has examined perceived social support and psychological resilience among older adults, and general emotion regulation and sleep quality among mixed middle-aged and older samples. The present study instead examined women aged 40–55 years who participated in several forms of mind–body exercise, including yoga, Pilates, Tai Chi, Baduanjin, and Qigong, and used nonspecific psychological distress rather than a single disorder or symptom category as the outcome. This focus broadens the relevance of research on psychological correlates of mind–body exercise to women aged 40–55 years who participated in several forms of mind–body exercise.

### 5.2. Practical Implications

This study offers preliminary directions for future intervention research concerning the mental health of women in midlife. The observed association between greater mind–body exercise participation and lower psychological distress suggests that future prospective and intervention studies could examine whether increasing participation in these activities is associated with subsequent changes in psychological distress. Unlike some forms of vigorous physical activity, practices including yoga, Pilates, Tai Chi, Baduanjin, and Qigong usually involve moderate movement and can be adjusted to suit different levels of physical ability. Future intervention studies could therefore evaluate the feasibility, acceptability, and psychological outcomes of community-based or workplace-based mind–body exercise programs for middle-aged women, rather than assuming their effectiveness on the basis of the present cross-sectional findings.

Second, cognitive reappraisal was statistically associated with both mind–body exercise participation and psychological distress. This finding generates the hypothesis that reappraisal may be a relevant target to examine in future intervention research. Future trials could test whether adding structured reappraisal or reflective components to mind–body exercise programs produces measurable changes in cognitive reappraisal and whether such changes are associated with subsequent psychological outcomes. Such components might include guided reflection on stressful experiences or exercises encouraging alternative interpretations of emotionally challenging situations. Their effectiveness, however, remains to be established empirically.

Third, the positive association between brooding and psychological distress suggests that brooding may represent another psychological factor worth testing in future interventions. Future studies could examine whether adjunctive components such as mindfulness-based attention training, relaxation practice, or group support produce reductions in brooding and whether these changes are associated with lower subsequent psychological distress. The present observational data do not establish that these components would be effective.

Overall, the findings generate testable hypotheses for future intervention research rather than evidence-based recommendations for program implementation. Future randomized or longitudinal studies could examine whether combining mind–body exercise with reappraisal-focused, mindfulness-based, relaxation, or social-support components produces changes in cognitive reappraisal, brooding, and psychological distress over time. Such studies are necessary before recommending these integrated approaches for routine practice.

### 5.3. Limitations

First, the cross-sectional design precludes conclusions about temporal ordering or causality. Although significant direct and indirect associations were observed among mind–body exercise, cognitive reappraisal, brooding, and psychological distress, these findings should not be interpreted as evidence of causal or temporal mediation. Reverse or bidirectional relationships are also possible; for example, women experiencing lower psychological distress may be more likely to participate more actively in mind–body exercise. Longitudinal and intervention studies are needed to clarify temporal and causal relationships.

Second, participants were recruited through convenience sampling from community-based exercise sites, which may have introduced selection bias and limited generalizability. Because all participants were regular mind–body exercise practitioners, the study did not include a non-practicing comparison group. Accordingly, the findings concern differences in participation within this selected group and cannot establish whether practitioners differ from non-practitioners. In addition, the sample combined heterogeneous modalities, including yoga, Pilates, Tai Chi, Baduanjin, and Qigong, which may differ in physical demands, attentional requirements, breathing practices, and meditative components. Future studies should use more representative samples, appropriate comparison groups, and, where feasible, modality-specific analyses.

Third, all variables were assessed simultaneously by self-report, creating potential recall, social desirability, and common-method biases. Although Harman’s single-factor test did not indicate a dominant factor, this test provides only a preliminary diagnostic, and common-method variance cannot be ruled out. Moreover, the PARS-3 scoring approach was adapted to heterogeneous mind–body activities and has not been specifically validated among middle-aged women, introducing additional uncertainty regarding the measurement of exercise participation. Future research should further validate this measure and incorporate objective, multi-source, or temporally separated assessments of exercise and psychological outcomes.

Finally, several potentially relevant characteristics were not collected, including exact age, exercise history and years of practice, general physical activity, and broader health information. Menstrual status, menopausal stage, menopausal symptoms, and hormone therapy were not assessed; therefore, participants cannot be classified as premenopausal, perimenopausal, or postmenopausal on the basis of chronological age alone. Psychiatric diagnoses, psychological treatment, psychotropic medication use, and other relevant clinical conditions were also not systematically assessed. Although sensitivity analyses adjusting for age, education level, and marital status showed that the principal structural associations remained substantively unchanged, residual confounding from unmeasured demographic, health, clinical, psychological, and exercise-related factors cannot be excluded. These unmeasured characteristics, together with the selected nature of the sample, further limit the external validity and generalizability of the findings. Future studies should assess a broader range of potential confounders and recruit more diverse samples.

## Figures and Tables

**Table 1:** Demographic characteristics of participants (N = 423).

Variable	Category	Survey (%)
Age	40–44	23.9
	45–49	58.4
	50–55	17.7
Education level	Junior high school or below	3.3
	High school or technical secondary school	44.0
	College or bachelor’s degree	48.2
	Master’s degree or above	4.5
Marital status	Married or cohabiting	87.2
	Unmarried, divorced, separated, or widowed	12.8
Employment status	Full-time employment	55.1
	Part-time or flexible employment	32.9
	Currently unemployed	12.1
Type of mind–body exercise	Yoga	32.9
	Pilates	37.4
	Tai Chi/Baduanjin/Qigong	20.1
	Other	9.7

**Table 2:** Reliability and validity.

Items	Factor Loadings	Cronbach’s α	Composite Reliability	AVE
Cognitive Reappraisal (CR)		0.899	0.899	0.597
CR1	0.782			
CR2	0.772			
CR3	0.754			
CR4	0.758			
CR5	0.774			
CR6	0.794			
Brooding (BR)		0.880	0.880	0.594
BR1	0.776			
BR2	0.755			
BR3	0.802			
BR4	0.772			
BR5	0.749			
Psychological Distress (PD)		0.896	0.896	0.590
PD1	0.777			
PD2	0.804			
PD3	0.787			
PD4	0.743			
PD5	0.745			
PD6	0.750			

**Table 3:** Descriptive statistics, correlations, and discriminant validity.

Construct	Mean	SD	Observed Range	MBE	CR	BR	PD
MBE	31.31	28.30	0–100	—			
CR	3.34	0.95	1.50–5.00	0.403 **	(0.773)		
BR	2.61	0.94	1.00–4.60	−0.373 **	−0.381 **	(0.771)	
PD	2.64	0.93	1.00–4.67	−0.374 **	−0.420 **	0.432 **	(0.768)

Note: MBE = mind–body exercise; CR = cognitive reappraisal; BR = brooding; PD = psychological distress. Values in parentheses on the diagonal are the square roots of AVE for the reflective latent constructs; off-diagonal values are Pearson correlation coefficients. MBE was treated as an observed composite variable and therefore has no AVE-based diagonal value. ** *p* < 0.01.

**Table 4:** Direct and indirect effects estimated using PROCESS Model 6.

Effect/Path	Effect	SE/BootSE	95% CI
Total effect: MBE → PD	−0.0123	—	—
Direct effect: MBE → PD	−0.0057	0.0015	[−0.0087, −0.0027]
Total indirect effect	−0.0066	0.0009	[−0.0084, −0.0048]
MBE → CR → PD	−0.0032	0.0007	[−0.0046, −0.0019]
MBE → BR → PD	−0.0024	0.0006	[−0.0035, −0.0014]
MBE → CR → BR → PD	−0.0010	0.0003	[−0.0015, −0.0005]

Note: MBE = mind–body exercise; CR = cognitive reappraisal; BR = brooding; PD = psychological distress. Indirect-effect confidence intervals were based on 5000 bootstrap samples.

**Table 5:** Sensitivity analysis adjusting for demographic covariates.

Structural Path	Primary Model β	Adjusted Model β	*p*
MBE → CR	0.430	0.430	<0.001
MBE → BR	−0.274	−0.283	<0.001
CR → BR	−0.309	−0.309	<0.001
CR → PD	−0.322	−0.320	<0.001
BR → PD	0.353	0.356	<0.001
Age → CR	—	−0.024	0.676
Age → BR	—	0.089	0.109
Age → PD	—	−0.026	0.630
Education → CR	—	0.029	0.605
Education → BR	—	0.086	0.125
Education → PD	—	−0.002	0.969
Marital status → CR	—	−0.008	0.867
Marital status → BR	—	0.035	0.449
Marital status → PD	—	−0.055	0.212

Note: MBE = mind–body exercise; CR = cognitive reappraisal; BR = brooding; PD = psychological distress. Values are standardized coefficients. The adjusted model included age, education level, and marital status as observed covariates, with direct paths from each covariate to CR, BR, and PD. Age was coded from 1 = 40–44 years to 3 = 50–55 years; education was coded from 1 = junior high school or below to 4 = master’s degree or above; marital status was coded as 0 = unmarried/divorced/separated/widowed and 1 = married/cohabiting.

## Data Availability

The data used to support the findings of this study are available from the corresponding author upon request.
